# Supplementary material for: Near-Infrared On-Site Evaluation (NOSE) Examination of EBUS/EUSb Samples—A New Method for Sample Adequacy Evaluation
Source: Diagnostics (Basel). 2024 Aug 28;14(17):1887. doi: 10.3390/diagnostics14171887 (PMC11394049; doi:10.3390/diagnostics14171887)
Supplement: Supplementary file 1 [file diagnostics-14-01887-s001.zip › diagnostics-3127991-supplementary.pdf]

Table S1. Whole set of acquired values

		Punch. nr.		1			2			3			4		
**Node no.**	**Initials**	**Birth year**	**R**	**D_{AFC}**	**SPR**	**R**	**D_{AFC}**	**SPR**	R	D_{AFC}	**SPR**	R	D_{AFC}	**SPR**	**Group finding**
1	R.B.	65			**N/A**[1]			**N/A**	1.14	0.037	**1.177**	1.063	0.28	**1.343**	2
2	A.K.	68			**N/A**			**N/A**	1.82	0.16	**1.98**			**N/A**	4
3	K.M.	55			**N/A**			**N/A**	1.69	0.113	**1.803**			**N/A**	2
4	V.M.	84	1.167	-0.024	**1.143**	1.412	0.08	**1.492**	1.43	0.01	**1.44**			**N/A**	2
5	J.N.	53	1.54	0.06	**1.6**	1.72	0.146	**1.866**	1.84	0.08	**1.92**			**N/A**	1
6	J.T.	45	1.62	0.09	**1.71**	1.71	0.24	**1.95**	2.11	0.19	**2.3**			**N/A**	1
7	M.Z.	58			**N/A**	1.6	0.105	**1.705**			**N/A**			**N/A**	2
8	J.H.	59	1.7	0.15	**1.85**	1.72	0.33	**2.05**	1.92	0.33	**2.25**	1.62	0.36	**2.08**	1
9	M.Š.	54	1.5	0.09	**1.59**	1.62	0.28	**1.9**	1.80	0.19	**1.99**	1.61	0.79	**2.4**	1
10	J.N.	55	1.7	0.21	**1.91**	1.64	0.18	**1.82**	1.63	0.26	**1.89**			**N/A**	1
11	H.S.	58	1.7	0.152	**1.852**	1.7	0.3	**2**	1.45	0.48	**1.93**			**N/A**	3
12	O.K.	47	1.64	0.17	**1.81**	1.63	0.23	**1.86**	1.61	0.23	**1.84**			**N/A**	1
13	H.Č.	42	1.66	0.03	**1.69**	1.63	0.04	**1.67**	1.66	0.08	**1.74**	1.68	0.07	**1.75**	4
14	R.M.	54	1.64	0.12	**1.76**	1.79	0.26	**2.05**	1.71	0.31	**2.02**	1.85	0.35	**2.2**	4
15	B.J.	56	1.8	0.21	**2.01**	1.79	0.36	**2.15**	1.49	0.18	**1.67**			**N/A**	2
16	B.J. (s.n)[2]		1.3	0.016	**1.316**	1.52	0.09	**1.61**			**N/A**			**N/A**	2
17	J.Š.	52	1.82	0.174	**1.994**	1.72	0.174	**1.894**	1.73	0.22	**1.95**			**N/A**	2
18	J.Š (s.n)		1.35	0.05	**1.4**	1.75	0.33	**2.08**	1.73	0.36	**2.09**			**N/A**	1
19	V.V.	67	1.4	0.12	**1.52**	1.39	0.14	**1.53**	1.58	0.18	**1.76**			**N/A**	1
20	V.V. (s.n.)		1.09	0.011	**1.101**	1.41	0.143	**1.553**	1.55	0.294	**1.844**			**N/A**	1
21	J.K.	46	1.21	0.028	**1.238**	1.69	0.34	**2.03**	1.61	0.24	**1.85**			**N/A**	1
22	R.K.	41			**N/A**	1.73	0.1	**1.83**	1.69	0.17	**1.86**			**N/A**	2
23	J.V.	67			**N/A**	1.74	0.5	**2.24**			**N/A**			**N/A**	1
24	J.K.	54	1.11	0.387	**1.497**			**N/A**			**N/A**			**N/A**	1
25	A.K.	73	1.092	-0.042	**1.05**	1.42	0.058	**1.478**	1.28	0.036	**1.316**			**N/A**	3
26	Č.S.	49	1.017	-0.001	**1.016**	1.30	0.022	**1.322**	1.46	0.055	**1.515**			**N/A**	3
27	Č.S. (s.n.)		1.55	0.048	**1.598**	1.72	0.043	**1.763**	1.71	0.075	**1.785**			**N/A**	3
28	J.V.	82	1.64	0.11	**1.75**	1.78	0.064	**1.844**	1.74	0.049	**1.789**			**N/A**	2
29	B.D.	63	1.12	0.01	**1.13**	1.39	0.072	**1.462**	1.42	0.072	**1.492**			**N/A**	2
30	K.U.	59	1.098	0.09	**1.188**	1.54	0.14	**1.68**	1.39	0.13	**1.52**			**N/A**	3
31	M.L.	51	1.32	0.19	**1.51**	1.77	0.23	**2**	1.82	0.37	**2.19**			**N/A**	2
32	M.L.(s. n.)	54			**N/A**	1.69	0.11		1.67	0.12	**1.79**	1.78	0.14	**1.92**	1
33	J.P.	39	1.14	0.03	**1.17**	1.57	0.15	**1.72**	1.62	0.22	**1.84**			**N/A**	1
34	V.M.	48	1.68	0.059	**1.739**	1.64	0.24	**1.88**	1.71	0.21	**1.92**			**N/A**	3
35	L.H.	44	1.22	0.12	**1.34**	1.49	0.11	**1.6**	1.56	0.19	**1.75**			**N/A**	1
36	F.O.	83	1.78	-0.048	**1.732**	1.87	0.015	**1.885**	1.55	0.05	**1.6**			**N/A**	3
37	A.P.	63	1.43	0.016	**1.446**	1.95	0.018	**1.968**	1.91	0.04	**1.95**			**N/A**	2
38	A.P. (s.n.)		1.38	0.097	**1.477**	1.74	0.31	**2.05**	1.97	0.43	**2.4**			**N/A**	1
39	T.J.	71			**N/A**				1.42	0.17	**1.59**	1.59	0.19	**1.78**	4
40	V.B.	91	1.088	-0.024	**1.064**	1.19	0.04	**1.23**						**N/A**	3

1

41	M.D.	49	1.55	0.152	**1.702**	1.67	0.33	**2**	1.52	0.36	**1.88**			**N/A**	3
42	Z.D.	59	1.59	0.07	**1.66**	1.57	0.18	**1.75**	1.59	0.32	**1.91**			**N/A**	1
43	O.B.	47	1.73	0.11	**1.84**	1.88	0.19	**2.07**	1.82	0.16	**1.98**			**N/A**	1
44	G.R.	82	1.089	-0.027	**1.062**	1.25	0.05	**1.3**	1.64	0.08	**1.72**			**N/A**	4
45	D.N.	57	1.19	0.013	**1.203**	1.44	0.098	**1.538**	1.37	0.11	**1.48**			**N/A**	2
46	D.N.(s. n.)		1.43	0.015	**1.445**	1.59	0.19	**1.78**	1.49	0.25	**1.74**	1.55	0.29	**1.84**	2
47	Č.D.	88	1.34	0.018	**1.358**	1.84	0.11	**1.95**	1.71	0.22	**1.93**			**N/A**	2
48	P.H.	57	1.11	0.039	**1.149**	1.73	0.038	**1.768**	1.71	0.07	**1.78**			**N/A**	1
49	M.L.	77	1.87	0.034	**1.904**	1.84	0.149	**1.989**	1.89	0.49	**2.38**	1.82	0.48	**2.3**	2
50	V.X.	69	1.57	0.14	**1.71**	1.59	0.39	**1.98**	1.69	0.37	**2.06**			**N/A**	1
51	H.J.	62	1.33	0.12	**1.45**	1.48	0.37	**1.85**	1.65	0.24	**1.89**			**N/A**	1
52	O.F.	78	1.23	-0.08	**1.15**	1.62	0.21	**1.83**	1.59	0.18	**1.77**			**N/A**	1
53	E.D.	72	1.67	0.085	**1.755**	1.65	0.15	**1.8**	1.67	0.11	**1.78**			**N/A**	2
54	W.R.	51	1.1	0.025	**1.125**	1.35	0.19	**1.54**	1.42	0.14	**1.56**			**N/A**	3
55	W.R. (s.n)				**N/A**				1.78	0.09	**1.87**	1.92	0.15	**2.07**	2
56	N.K.	87	1.82	0.031	**1.851**	1.64	0.14	**1.78**			**N/A**			**N/A**	2
57	E.S.	62	1.67	0.082	**1.752**	1.72	0.084	**1.804**	1.71	0.27	**1.98**			**N/A**	4
58	J.K.	55	1.24	0.12	**1.36**	1.41	0.11	**1.52**	1.82	0.11	**1.93**			**N/A**	1
59	A.B.	45	1.18	0.013	**1.193**	1.53	0.183	**1.713**	1.62	0.22	**1.84**			**N/A**	4
60	T.P.	48	1.35	0.011	**1.361**	1.73	0.175	**1.905**	1.79	0.39	**2.18**	1.99	0.35	**2.34**	1

[1]this puncture was not analyzed.

[2]second node puncture .
